# Severe, Complicated Pancreatitis With an Unclear Etiology

**DOI:** 10.7759/cureus.39011

**Published:** 2023-05-14

**Authors:** Brian Nohomovich, Ali Shah, Neil Hughes

**Affiliations:** 1 Internal Medicine, Western Michigan University Homer Stryker M.D. School of Medicine, Kalamazoo, USA

**Keywords:** idiopathic pancreatitis, lams, pseudocysts, pancreatitis, semaglutide, covid-19

## Abstract

Acute pancreatitis is an inflammatory process. There can be many causes of pancreatitis, which include alcohol or gallstones but can also be due to hypercalcemia, infections, or hypertriglyceridemia. Most cases of pancreatitis are mild and without complications. Severe cases of pancreatitis can cause complications, including organ failure. Pseudocysts are a rare complication of pancreatitis and may require management. We present a patient with severe acute pancreatitis with organ failure admitted to the intensive care unit, stabilized, and required subsequent management of a pseudocyst with cystogastrostomy with a lumen-apposing metal stent. The patient subsequently improved and is doing well today. Herein, we present an acute severe pancreatitis case report with an extensive workup complicated by pseudocyst development. We review pancreatitis causes, including rare causes and management.

## Introduction

Acute pancreatitis is an inflammatory process with an incidence estimated at 20 to 40 people per 100,000 per year, with approximately 15% to 25% of all cases of acute pancreatitis developing severe pancreatitis [1,2]. The severity of pancreatitis is based on demographic factors, local complications, and organ failure [1]. Pancreatitis severity is graded on the Atlanta classification [3], which was noted to have limitations and subsequently revised [4]. The revised Atlanta classification has two categories based on parenchymal or peripancreatic necrosis. The severity of pancreatitis is then based on whether it is mild, moderate, or severe. Mild pancreatitis lacks the absence of organ failure and local or systemic complications. Moderately severe acute pancreatitis has transient organ failure or local or systemic complications that do not persist past 48 hours. Severe acute pancreatitis is defined as persistent organ failure that may involve multiple systems [4]. Pancreatitis with persistent organ failure occurs in less than 5% of cases [5]. Organ failure is due to an imbalance between inflammatory and anti-inflammatory responses [6]. Acute pancreatitis occurs through an inciting event that causes damage to the pancreas, releasing pancreatic enzymes into circulation, which could further damage the pancreas and initiate an inflammatory response [6]. In the United States, gallstones account for 40% to 70% of cases of acute pancreatitis [7]. Alcohol use accounts for 25% to 35% of cases of acute pancreatitis in the United States [8]. Additional considerations include hypertriglyceridemia (> 1000 mg/dl) [9], hypercalcemia [10], medications [11], and infections [12]. As a complication of acute pancreatitis, a pseudocyst may develop over 4-6 weeks following the initial episode of pancreatitis. The incidence of pseudocyst development is 1.6% to 4.5% [13]. Most cases of pseudocyst development are likely alcohol-induced [14]. A pseudocyst develops from a pancreatic ductal leak following an insult leading to extravasation of pancreatic secretions into the surrounding tissue [15]. If the fluid collection persists, a fibrous granulation wall will appear after 4-6 weeks [15]. We present a patient with acute, severe pancreatitis of unclear etiology complicated by two enlarging pseudocysts requiring stent placement.

## Case presentation

Our patient is a female in her sixties with a past medical history of rheumatoid arthritis on chronic 5 mg twice daily prednisone, major depressive disorder on duloxetine, and type 2 diabetes mellitus (T2DM), for which she was switched from metformin to semaglutide six weeks prior. There are no known medication allergies. She lives independently, is a former smoker, and denied recent alcohol use.

Four weeks before presentation at our hospital, she was hospitalized for one week due to COVID-19 pneumonia and was treated with supportive care and discharged without complications. Two weeks prior, she was admitted to an outside hospital for diabetic ketoacidosis (DKA) and acute pancreatitis. Lipase > 4000 IU/L (25-78 IU/L) computerized tomography (CT) of the abdomen and pelvis with contrast performed at admission showed a small, developing pseudocyst behind the stomach. The patient was started on ampicillin-sulbactam due to persistent leukocytosis. Semaglutide was held at admission by the outside hospital due to acute pancreatitis. Due to persistent abdominal pain, the CT of the abdomen and pelvis with contrast was repeated three days later and showed enlargement of the pseudocyst to approximately 7 cm in size with ascites. One week after initial admission, the pseudocyst was 13 cm in size. Ultrasound of the right upper quadrant was negative for cholelithiasis. Magnetic resonance imaging (MRI) of the abdomen and pelvis showed hepatic steatosis, pleural effusions, and anasarca.

The patient was transferred to our hospital for further management of acute pancreatitis and drainage of the pseudocyst. The patient reported constant 10/10 epigastric pain, shortness of breath, generalized weakness, nausea, and constipation. Initial vitals showed a temperature of 36.5°C, a heart rate of 96 beats per minute, a respiratory rate of 16 breaths per minute, a blood pressure of 126/62 mmHg, and SpO2 of 98% on a four-liter nasal cannula. She does not use oxygen at baseline. The patient was alert and orientated with generalized pallor, diffuse crackles bilaterally on lung fields, and tenderness to palpation in the epigastric region but without signs of peritonitis, perforation, or rebound tenderness. She had bilateral lower extremity pitting edema. She had a prior right foot trans-metatarsal amputation and a swan neck deformity of multiple hand digits. Her skin was warm and dry, without rashes. No focal deficits were seen on the neurological exam. See Table 1 for the complete labs. Regarding the workup for acute pancreatitis, serum triglycerides were within normal limits, serum calcium was low, IGG4-levels for autoimmune pancreatitis were normal, there were no gallstones on imaging, the patient denied alcohol use, and COVID-19 PCR was negative. 

**Table 1:** Admission labs Unless noted above, all other serum electrolytes, serum creatinine, glomerular filtration rates, coagulation studies, and low-density lipoprotein cholesterol were within normal limits. IGG-4 levels were normal.

Lab	Value	Reference Range
Hemoglobin	8.2	12.0-15.0 g/dL
Hematocrit	23.8	36-46 %
Mean Corpuscular Volume	89.3	83-101 fL
White blood cell count	33.3	4.5-11.0 K/uL
Absolute Neutrophil count	29.9	1.8-7.7 K/uL
Platelet count	640	150-400 K/uL
Serum Sodium	130	135-145 mmol/L
Serum Chloride	98	101-111 mmol/L
Serum Calcium	7.8	8.4-10.3 mg/dL
Serum Calcium ionized	3.7	4.25-4.6 mg/dL
Serum Magnesium	1.7	1.8-2.5 mg/dL
Serum Glucose	68	70-99 mg/dL
Blood Urea Nitrogen	22	8-21 mg/dL
Serum Alkaline Phosphatase	395	45-115 IU/L
Serum Albumin	1.8	3.5-4.8 g/dL
Serum lipase	137	25-78 IU/L
Total cholesterol, serum	89	112-199 mg/dL
High-density lipoprotein cholesterol, serum	13	40-75 mg/dL
Triglycerides, serum	112	0-149 mg/dL

The patient developed moderate ascites and underwent therapeutic paracentesis with the removal of 1800 ml of exudative ascites with elevated amylase levels. Ascitic labs showed ascitic albumin of 1.5 g/d, corresponding serum albumin of 2.1 g/dl (serum albumin ascites gradient of 0.6 g/dl), and ascitic protein of 2.7 g/dl. Ascitic cultures were negative for infection.

**Figure 1:**
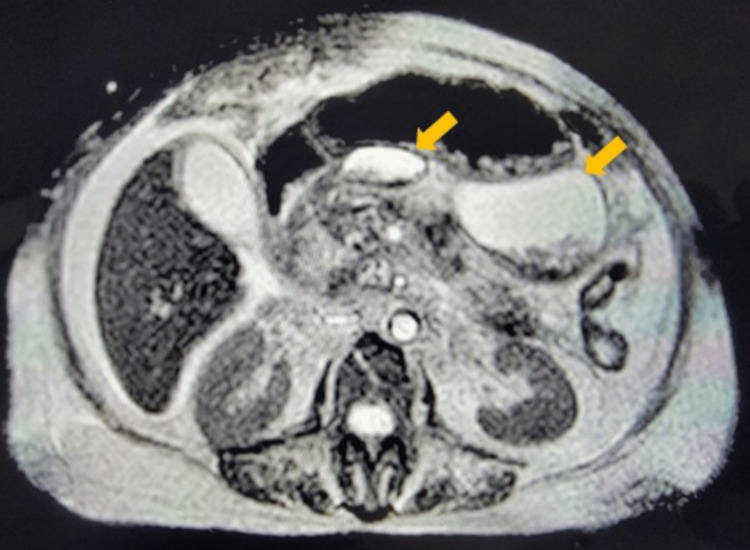
Magnetic resonance cholangiopancreatography Two large pseudocysts are present in the head and tail of the pancreas (yellow arrows).

She continued to decompensate through the course of her hospital stay. She had a cardiac arrest and was successfully resuscitated and intubated. She was transferred to the intensive care unit (ICU). Magnetic resonance cholangiopancreatography (MRCP) (Figure 1) showed no pancreatic duct injury contributing to pancreatic ascites. The complicated pseudocysts within the head of the pancreas increased in size to 4.2 x 3.6 cm, while the pseudocyst along the posterior margin of the stomach's greater curvature showed layering fluid/debris with an increase in size to 6.4 x 7.7 x 10.2 cm (Figure 1). Dark brown fluid was aspirated percutaneously from the left subdiaphragmatic region to assess the peripancreatic fluid collection, which was subsequently negative for infection. Her ICU course was complicated by septic shock from *Pseudomonas aeruginosa* and hospital-acquired pneumonia treated with meropenem, vancomycin, anidulafungin, and norepinephrine for blood pressure support. She had persistent leukocytosis from 19 to 49 (4.5-11.0 K/uL) throughout the hospital stay. Electrolytes were corrected, and glucose levels were under control with basal and sliding-scale insulin.

The patient was transferred to an outside facility for a higher level of care. At the outside facility, the patient underwent cystogastrostomy with a lumen-apposing metal stent (LAMS) and necrosectomy to drain the pseudocyst along the posterior wall of the stomach's greater curvature, followed by LAMS removal and replacement with plastic stents for continuous internal drainage. A percutaneous biliary drainage tube was placed due to the slowing of the biliary drainage secondary to the mass effect of a complicated pancreatic pseudocyst within the head of the pancreas. The patient subsequently improved and was discharged to a long-term acute care facility for rehabilitation. She was continued on basal-bolus insulin until she could be seen by her primary care provider. 

## Discussion

Pseudocysts can develop from acute or chronic pancreatitis with severe necrosis [15]. Symptoms due to pseudocysts include abdominal pain, often worse with eating, weight loss, gastric outlet obstruction, obstructive jaundice, and pancreatic duct leakage. Pancreatic duct leakage can lead to ascites, as seen in our patient, or fistula formation. Infection may also develop, which is an absolute indication for drainage [15]. Traditionally, a size cut-off of > 6 cm or persistence of the pseudocyst was used as an indication for drainage [15]. Still, some patients may remain asymptomatic and stable with a low risk of infection, bleeding, or rupture [16]. Enlargement of pseudocysts is an indication for drainage, as was the case in our patient [17]. After careful history-taking, coagulation studies should be ordered. A CT of the abdomen with contrast should be done to assess the location and relation to nearby structures like the stomach, duodenum, and vasculature. Endoscopic ultrasound or magnetic resonance imaging can also assess the presence of solid debris. Endoscopic ultrasound allows for a definitive diagnosis using ultrasound features, aspiration, and subsequent analysis of cyst contents [15]. Walled-off pancreatic fluid collections result from pancreatitis at least four weeks prior [15]. The patient subsequently developed pancreatic ascites, likely secondary to the pseudocysts. Conservative management of pancreatic ascites was initiated, including holding oral tube feeds and starting octreotide and parenteral nutrition [18]. Conservative management has a failure rate of as high as 60% and a mortality rate of 17% [19]. Endoscopic ultrasound-guided transmural drainage is adequate for most patients with pancreatic pseudocysts abutting the stomach or duodenum [20] and is likely more effective than an endoscopic approach [21]. In severe cases, as with our patient, cystogastrostomy with LAMS and necrosectomy [22] might be required to drain the pseudocyst and facilitate biliary drainage.

The patient initially presented two weeks ago to an outside facility with acute pancreatitis and was found to have an enlarging immature pseudocyst at that time. She endorsed some mild abdominal discomfort that had been ongoing for a couple of weeks before the presentation at the outside facility. Pancreatitis workup for common causes was negative in our patient. Gallstones were not identified with the right upper quadrant ultrasound or CT. The patient denied alcohol use. Serum calcium and triglycerides were within normal limits. In discussing the history with the patient, two events fit within the timeframe of pseudocyst development. The first was an infection with COVID-19, which occurred four weeks prior and coincided with subsequent abdominal discomfort. The timeframe would fit pseudocyst development. The data on acute pancreatitis from COVID-19 is mixed. A systematic review of 66 cases of concomitant COVID-19 and acute pancreatitis identified that patients with both conditions required higher levels of care and complications from acute pancreatitis [23]. It has been reported that 17% of patients with COVID-19 have elevated amylase/lipase levels, and noted that if pancreatitis was present, it was mild [24]. The significance of this finding is unclear, as amylase/lipase levels can be elevated due to other disease processes like gastroenteritis or gastrointestinal symptoms, which are common with COVID-19. In a retrospective study, 32/11883 (0.3%) of patients with COVID-19 also had acute pancreatitis [25], but no clear association was drawn.

Interestingly, the receptor for viral entry, angiotensin-converting enzyme 2 (ACE2) receptors, was highly expressed in the pancreas [26], suggesting a possible mechanism for viral infection. COVID-19 RNA has been detected in a pancreatic fluid collection [27]. It is unclear if this was a genuinely infectious agent or retrograde contamination. COVID-19 testing of fluid collections is not routinely done, but it is an interesting finding given the presence of ACE2 receptors in the pancreas. Three other case reports have implicated COVID-19 in pseudocyst formation [27-29]. In each reported COVID-19 and pseudocyst formation case, the patient had a current COVID-19 infection confirmed by polymerase chain reaction testing. Our case is unique because the patient presented with the pseudocyst four weeks after her initial COVID-19 infection, which is inconsistent with the reported case results.

Additionally, the patient was hospitalized for DKA two weeks before presentation at our hospital and two weeks after her COVID-19 diagnosis. Our patient was never hospitalized for DKA before. We suspect underlying pancreatic dysfunction that developed after her discharge following the COVID-19 infection and the development of DKA. COVID-19 can cause hyperglycemia or DKA through oxidative stress, a cytokine storm causing insulin resistance, and direct β-cell damage [30]. At the time of presentation with DKA, the patient was noted to have acute pancreatitis. The incidence of acute pancreatitis with DKA is estimated at 10% to 15% [31]. It is difficult to discern if DKA is a complication or the cause of acute pancreatitis. Acute pancreatitis is directly associated with DKA and hyperlipidemia [32-34]. Our patient had normal lipid levels. We suspect the DKA was likely a complication of pancreatic inflammation and β-cell dysfunction at the time.

She started semaglutide six weeks before her presentation at our hospital and reportedly tolerated the medication well. Semaglutide is a glucagon-like peptide-1 analog that increases β-cell insulin secretion and improves glycemic control [35] and is approved for treating T2DM [36]. Semaglutide reduces cardiovascular risks in persons with T2DM and cardiovascular disease [37] and can help sustain weight loss [38]. The most common side effects are nausea, vomiting, diarrhea, or constipation [38]. It is associated with severe gastrointestinal adverse events, gallbladder-related disorders, and hepatobiliary disorders [38,39]. Acute pancreatitis is a rare side effect, with a recent study showing an incidence of 0.2% [38]. However, post-marketing surveillance identified that acute pancreatitis from semaglutide is likely a severe adverse event [40]. The time to onset for acute pancreatitis development after the first dose of semaglutide is 23 days, with an interquartile range of two to 92 days [40]. Our patient received their first dose of semaglutide 42 days before presentation, which falls well within the interquartile range. Several studies have not substantiated the association between semaglutide and acute pancreatitis [41-44]. Further research is needed to investigate any possible causal mechanism between semaglutide and pancreatitis. There have been no case reports of pseudocyst development following a case of acute pancreatitis that was attributed to semaglutide.

## Conclusions

The etiology of this case of severe pancreatitis is unclear, but it is likely attributable to either COVID-19 or semaglutide. The case highlights the morbidity that acute pancreatitis can cause and the resulting complications. Clinicians should consider common causes of acute pancreatitis, like alcohol and gallstones. A history from the patient should be obtained, noting recent medication changes or infections. A workup should be ordered to rule out common causes of acute pancreatitis, including a lipid panel, serum calcium levels, and at least a right upper quadrant ultrasound. Pseudocysts can develop in a case of acute pancreatitis and should be given time to mature if possible. Most pseudocysts can be managed conservatively with pain management and observation. Complicated pseudocysts may require surgical involvement. Further research is needed to investigate the pathophysiology behind less common causes of acute pancreatitis.
